# Acute Postexercise Change in Circulating Irisin Is Related to More Favorable Lipid Profile in Pregnant Women Attending a Structured Exercise Program and to Less Favorable Lipid Profile in Controls: An Experimental Study with Two Groups

**DOI:** 10.1155/2019/1932503

**Published:** 2019-02-28

**Authors:** Anna Szumilewicz, Aneta Worska, Magdalena Piernicka, Agnieszka Kuchta, Zbigniew Jastrzębski, Łukasz Radzimiński, Marta Kozłowska, Katarzyna Micielska, Ewa Ziemann

**Affiliations:** ^1^Department of Fitness and Strength Conditioning, Gdansk University of Physical Education and Sport, Poland; ^2^Department of Clinical Chemistry, Medical University of Gdansk, Poland; ^3^Department of Biomedical Health Basics, Gdansk University of Physical Education and Sport, Poland; ^4^Department of Physiology and Pharmacology, Gdansk University of Physical Education and Sport, Gdansk, Poland; ^5^Department of Anatomy and Anthropology, Gdansk University of Physical Education and Sport, Gdansk, Poland

## Abstract

**Introduction:**

The blood irisin concentrations may be affected both by exercise and pregnancy. We aimed to determine acute responses in serum irisin after a single exercise session and relationships between exercise-induced changes in this hormone and lipid profile in pregnancy.

**Material and Methods:**

It was an experimental study in 20 Caucasian women in normal pregnancy (age 30 ± 3 years, 28 ± 6 weeks of gestation; mean ± SD). Participants were assigned to training (*n* = 8) and control groups (*n* = 12). Before the experiment, women from the training group attended a structured exercise program 3 times a week for 6 weeks. Blood samples were collected before and 30 minutes after a single bout of 60-minute moderate- to high-intensity exercise to determine serum levels of irisin, insulin, glucose concentration, and lipid profile.

**Results:**

At baseline, we recorded slightly lower irisin levels in the training group compared to controls (12.2 ± 2.4 and 13.9 ± 3.3 ng · ml^−1^, respectively). Only in the training group all women presented increase in irisin levels after exercise (on average by 14%); and this change was statistically significant (*p* = 0.002). In the controls, we found positive significant relationships between postexercise irisin change and low-density lipoproteins (*R* = 0.594; *p* = 0.04) and total cholesterol (*R* = 0.734; *p* = 0.006). Surprisingly, in the training group, these relationships were also significant but inverse (*R* = −0.738 and *p* = 0.036; *R* = −0.833 and *p* = 0.01, respectively).

**Conclusions:**

Training and control pregnant women responded differently to a single exercise session, both in the postexercise change in irisin and its relationship to the blood lipids. Only in the training group we observed the postexercise increase in irisin, which was related to more favorable lipid profile. Systematic prenatal physical activity may optimize the postexercise irisin response and lipid metabolism regulated by this hormone. Therefore, exercise programs should be promoted in pregnant women and obstetric care providers.

## 1. Introduction

According to current recommendations, pregnant women should perform at least 150 minutes per week of moderate-intensity aerobic activity to improve or maintain cardiorespiratory fitness and reduce the risk of obesity and associated comorbidities [1]. Several authors have found that supervised physical exercise during pregnancy had positive effect on maternal lipids [2, 3]. In turn, sedentary behavior in pregnant women was associated with unfavorable lipid profile [4, 5]. The increase in lipids in pregnancy is a physiological condition, principally because of changes in hormones during the trimesters of gestation. However, pathological levels of cholesterol and triglycerides, called dyslipidemia, are associated with gestational diabetes mellitus, preeclampsia, preterm birth and other adverse outcomes such as low birth weight, or risk of macrosomia [6]. Therefore, preventing dyslipidemia during gestation, inter alia through regular physical activity, seems important. Through this study, we wanted to better understand the mechanism of maintaining lipid homeostasis in pregnant women through regular exercise.

Irisin is an exercise-induced myokine, which plays an important role in lipid homeostasis, affecting the browning of white adipose tissue and increasing energy expenditure using lipids. Thus, it might be a therapeutic hormone for noncommunicable diseases. It is a terminal product of proteolytic cleavage of fibronectin type III domain-containing protein (FNDC5) [7]. According to Huh et al. [8], increased irisin can directly modulate muscle metabolism through the activation of adenosine monophosphate-activated protein kinase (AMPK), which inter alia inhibits cholesterol and triglyceride synthesis and stimulates skeletal muscle fatty acid oxidation [9]. Still, the processes linking irisin and lipid profile are still unclear. In healthy nonpregnant women, some authors observed inverse associations between irisin and serum lipids [9–11]. In other reports, these correlations were positive [12, 13]. Unfortunately, in most works, participant's physical activity was neither analyzed nor reported. Benedini et al. found that the relationship between irisin and lipids in blood substantially varies depending on physical activity patterns [14].

In pregnancy, the irisin metabolism seems to change significantly. In some studies, the rise of irisin throughout gestation in healthy women has been reported [15–17]. Irisin mRNA expression in placenta is low as compared to human muscle and is not a major contributor to circulating irisin in gestation [9, 18]. A few authors recorded that in general population of pregnant women irisin was positively correlated with total cholesterol (TC) [18, 19] and also with low-density lipoproteins (LDL), high-density lipoproteins (HDL), and triglycerides (TG) [18]. In contradiction to these results in our previous study in regularly exercising pregnant women, we observed neither the rise of baseline serum irisin with the development of pregnancy nor its positive relationship with serum lipids [20]. In this study, first, we aimed to evaluate acute responses in circulating irisin after a 60-minute exercise session in pregnant women regularly participating in a structured exercise program for six weeks in comparison to healthy pregnant controls. Second, we wanted to investigate whether there is a relationship between postexercise change in serum irisin and lipid profile in pregnancy.

## 2. Materials and Methods

### 2.1. Study Design and Participants

It was an experimental study with two groups. We tested 20 Caucasian nulliparous healthy women in normal pregnancy (age 30 ± 3 years, 28 ± 6 weeks of gestation; mean ± SD), who volunteered for blood collection before and after participating in a single exercise session. In the training group, there were 8 women, who completed a 6-week cycle of structured prenatal exercise with the exercise frequency of three times a week. The control (nontraining) group consisted of twelve age-matched women who came to us to start this six-week exercise program, regularly run at our university.

**Figure 1:**
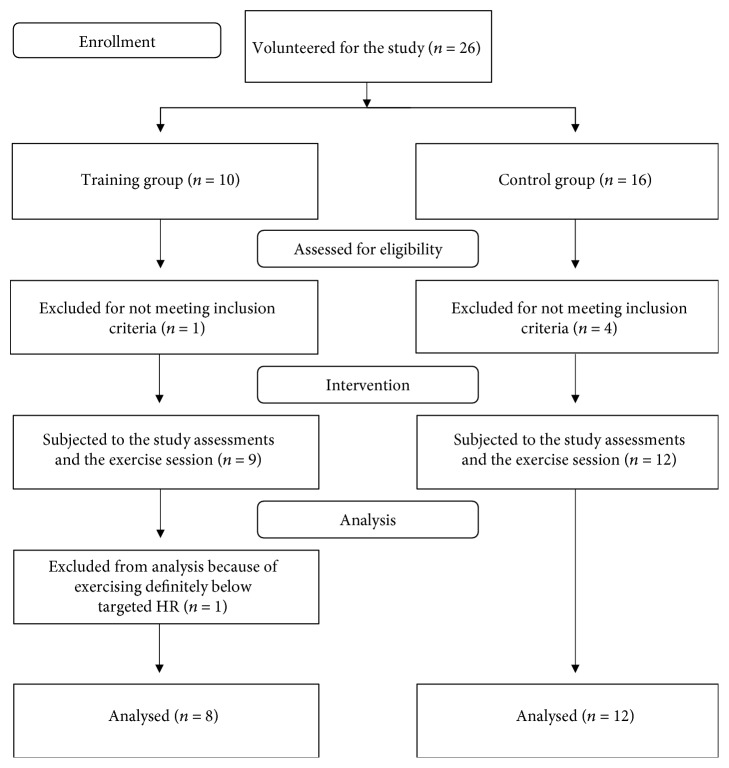
The flow of participants through the study.

All women volunteered for the study by completing the electronic form available on the website of the experiment. The eligibility criteria were single pregnancy, a positive assessment of woman's health, and normal pregnancy, including normal prepregnancy BMI and normal gestational weight gain, confirmed by an obstetric care provider. In the study, we included only women who reported recommended level of physical activity before the conception. From the control group, we additionally expected the declaration that they had not participated in any structured exercise program from the beginning of pregnancy to the experiment enrolment. Women's health condition and the course of pregnancy were assessed on the routine prenatal consultation, according to the national law. The prepregnancy level of physical activity was assessed through the initial online interview. Exclusion criteria were history of miscarriages over 12 weeks of gestation and/or more than two successive miscarriages in the first trimester and any contraindications to exercise. The flow of participants through the study is presented in Figure 1.

We conducted the study in the Laboratory of Physical Effort and Genetics in Sport at Gdansk University of Physical Education and Sport (AWFiS) in Poland between January and March 2016. The study was performed according to the principles of the Helsinki Declaration and project's approval of the Bioethics Commission in Gdansk (KB–8/13 and KB–22/15). The participants signed the informed consent before testing.

### 2.2. Assessment of the Exercise Capacity

Three days before the experiment all women underwent an exercise test on a cycloergometer with electronically regulated load (Viasprint 150P). In order to establish the maximum uptake of oxygen, we have used stationary respiratory gas analyzer (Oxycon Pro, Erich Jaeger GmbH, Germany). It was calibrated prior to each test according to manufacturer's instructions. Breath-by-breath data were averaged to provide a data point for each 15-second period.

The test started with a 5-minute adaptation phase when women sat in a chair. There followed a 4-minute warm up with a relative load of 0.4 W/kg of body mass. After the warm up, the load increased by 0.2 W/kg every minute up to refusal. Before the experiment, we instructed women to use the 0-10 Borg Perceived Exertion Scale [21]. They were allowed to stop the test at any time. As women's maximal effort, we treated the test results when they achieved the perceived exertion level of 9 or 10 and the value of Respiratory Exchange Ratio (RER) was above 1. After the test, the study participants rested for 3 minutes sitting in a chair. The highest oxygen uptake achieved during the maximum effort and maintained for 15 seconds was taken as maximal oxygen capacity (VO_2 max_).

Based on the RER value, we have set heart rate zones for exercise sessions. The lower heart rate limit corresponded to the RER value of 0.85. The upper heart rate limit was set at the RER value equal to 1, which corresponds to the maximal lactate steady state (MLSS) [22]. MLSS represents the exercise intensity above which a continuous increase in blood lactate is unavoidable and refers to the term “anaerobic threshold” [23]. Keeping heart rate between these thresholds was to provide participants with aerobic exercise and optimize cardiopulmonary fitness [24]. Because in some women the RER values may be higher in pregnancy due to shifting metabolic energy substrates [25], we also analyzed carefully HR curves. We decided for aerobic exercise, because apart from numerous benefits typical for general populations, it compensates for the physiological changes in woman's body induced by pregnancy [26].

### 2.3. Blood Collection and Analysis

Before and after the exercise session, blood samples were taken from the antecubital vein into the vacutainer tubes with EDTAK_2_. For the purpose of assessing glucose level, blood was taken into the vacutainer tubes with sodium fluoride. In order to control glucose alternation, glycated hemoglobin was also determined. At the first time point, the blood samples were taken in a fasting condition. After the first blood collection, all women ate the same light breakfast and after 50-60 minutes of rest they started to exercise for 60 minutes. Before the first blood collection, the training women had about 69 hours of break after the last bout of exercise (they finished their last exercise session on Friday at 10:30 a.m. and the blood samples were taken between 7:30 and 8:00 a.m. on the following Monday).

The second blood collection was 30 minutes after exercise. Immediately following the blood collection, one portion of the sample was transferred to centrifuge tubes containing aprotinin (catalog no. RK-APRO) from Phoenix Pharmaceuticals Inc. The final concentration of aprotinin was 0.6 Trypsin Inhibitor Unit/1 ml of blood. The samples were centrifuged at 2000 g for 10 min at 4°C. The separated serum samples were frozen and kept at –70°C until later analysis. Quantification of serum irisin was based on a competitive enzyme immunoassay and the assay kits were purchased from Phoenix Pharmaceuticals Inc. (catalog no. EK 067-29). Details on the ELISA have been described elsewhere [27]. The dilution of sample was 1 : 1. The intra-assay coefficients of variability (CVs) and interassay CVs reported by the manufacturer were 4%–6% and 8%–10%, respectively.

Insulin was determined also by enzyme immunoassay methods using commercial kit DiaMetra (DCM076-8). The variability within assay was ≤5%. The hematological measurements were performed using conventional methods with a COULTER® LH 750 Hematology Analyzer (Beckman-Coulter, USA). The serum concentrations of the total cholesterol (TC), high- and low-density lipoproteins (HDL, LDL), and triglycerides (TG) were determined with commercial kits using enzymatic methods (Alpha Diagnostics, Poland). LDL cholesterol was calculated using the Friedewald formula. Glucose was assessed using analyzer Cobos 6000.

### 2.4. Prenatal Exercise Session as Experimental Intervention

Pregnant women participated in a single moderate-to-high intensity exercise session, designed by the principal researcher of the study according to the available guidelines [1, 28, 29]. The exercise session was held from 9:30 to 10:30 a.m. at the sport facility of Gdansk University of Physical Education and Sport. It consisted of warm up and aerobic part in the form of high-low impact aerobics choreography with music (25 min), resistance exercises (25 min), pelvic floor muscle exercises, stretching and breathing exercises, and relaxation (10 min). To maintain proper intensity of exercise during the aerobic part, we used heart rate monitors (Polar RS400, Finland) with individually adjusted heart rate zones. Before the experiment, we instructed women how to observe changes in their heart rate and to keep it within the stated ranges. Additionally, they monitored the exercise intensity based on the Rating of Perceived Exertion Scale (RPE) and the “talk test” [1]. In the strengthening part, women performed nine exercises for each muscle group in two sets of 12-16 repetitions, with a break of 30 s between sets. We instructed the participants to perform the repetitions until they felt unpleasant soreness of the targeted muscles. No equipment was used during exercises and only resistance of own body was applied.

The session was conducted by a certified Pregnancy and Postnatal Exercise Specialist whose competences met the European educational standard for this profession [30]. She was informed of the aim of the study and trained in terms of monitoring and maintaining the desired intensity of exercise among participants (inter alia by using rest breaks or implementing jumps and optional repetitions). The principal researcher was checking the quality of the exercise during the entire session. We have not observed any adverse outcomes of implementing this exercise intervention.

Before the experiment, women from the training group participated in such exercise sessions 3 times a week for 6 weeks.

### 2.5. Sample Size Estimation and Statistical Analysis

We have not calculated the sample size in this study. We realized that experimental interventions in pregnancy may seem controversial for women and our research procedure required both tiring exercise capacity assessment and a double blood collection before and after an intense exercise session. Therefore, in this study, we have examined all available pregnant volunteers, meeting inclusion criteria.

Statistical analysis was performed using the Statistica software package (STATISTICA 13.1, StatSoft Poland) and GraphPad Prism 4.03 software. Descriptive characteristics of variables, after analyzing their distribution with Shapiro-Wilk test, were expressed using mean ± standard deviation (SD) or medians with 25th and 75th percentiles. To compare the values of selected variables between groups before the intervention, we used independent sample *t*-test or Mann-Whitney test. Post-preexercise changes in irisin in each group were analyzed using *t*-test for repeated measures. Univariate correlations were assessed using Spearman coefficients. The *p* value obtained of less than 0.05 was considered statistically significant.

## 3. Results

In Table 1, we presented the characteristics of the study participants. Both groups presented similar values in terms of age, physical fitness, and exercise heart rate zones. All women were in the second half of their pregnancy. However, due to the study design (see Methods), the training group was in a slightly higher week of gestation (Table 1). Correspondingly to higher pregnancy, they also presented higher BMI values compared to controls. Nevertheless, all women had normal prepregnancy BMI and normal gestational weight gain, which were our inclusion criteria to this experiment.

In both groups, the levels of all analyzed markers of lipid and glucose metabolism corresponded to the reference values for a given stage of pregnancy [31]. Most likely, the lower week of gestation in controls determined their significantly lower baseline values of glycated hemoglobin (HbA1c), triglycerides (TG), total cholesterol (TC), and low-density lipoproteins (LDL), compared to the training group (Table 2).

Before the exercise session, we recorded insignificantly lower concentrations of circulating irisin in the training group compared to controls. The irisin levels were not correlated with week of gestation neither in the training (*R* = 0.021; *p* = 0.96) nor in the control (*R* = −0.162; *p* = 0.62) groups.

**Figure 2:**
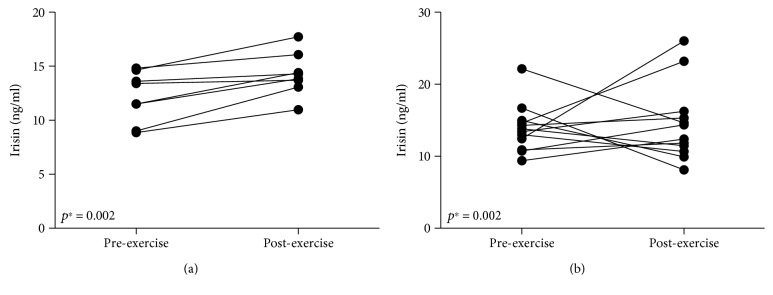
The irisin concentration before and after exercise session in training women (a) and control group (b); ^∗^Student's *t*-test for repeated measures.

An interesting outcome is that all women in the training group presented increase in irisin levels after the exercise session (on average by 14%; 2.09 ± 1.28 ng · ml^−1^); and the rise of irisin for this group was statistically significant (*p* = 0.002) (Figure 2). On the contrary, the control group responded in diverse ways. In seven controls, we observed higher postexercise irisin concentrations compared to baseline (on average by 37%; 4.79 ± 4.63 ng · ml^−1^), whereas in five women the irisin level dropped after exercise (on average by -32%; -5.16 ± 2.87 ng · ml^−1^).

**Figure 3:**
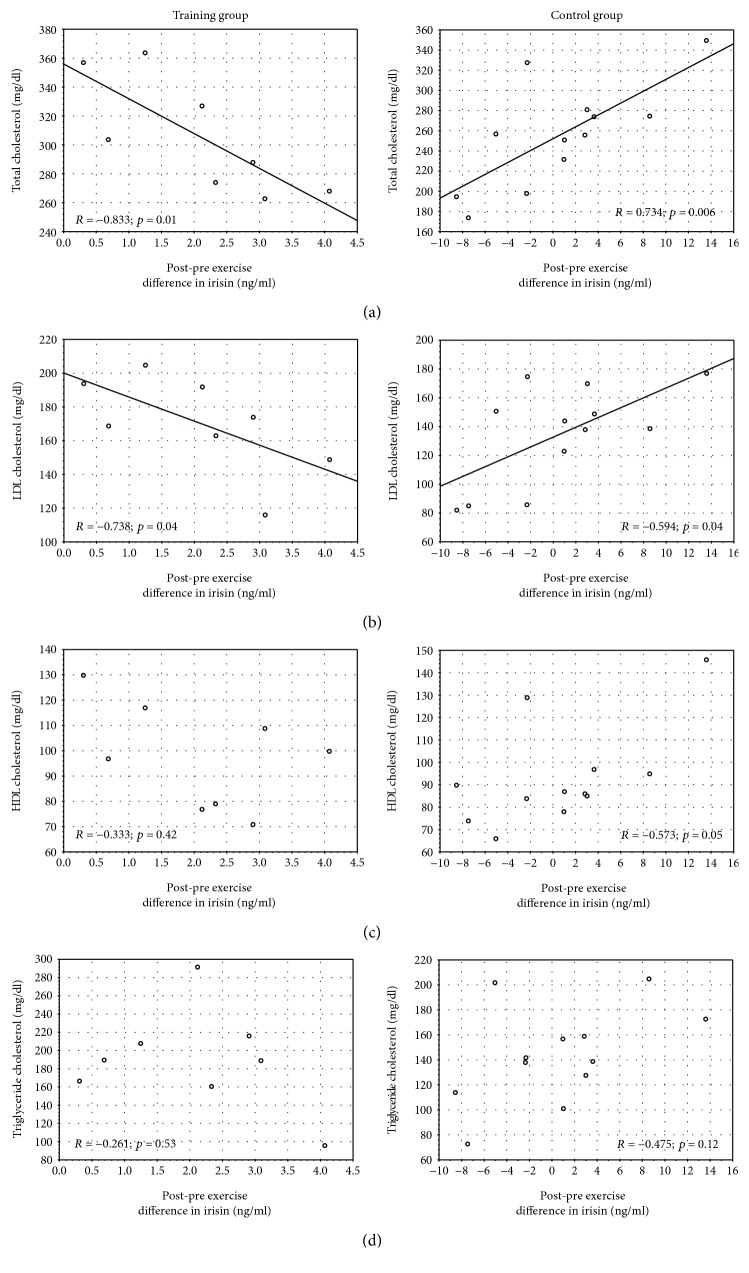
The correlation between the changes in irisin concentration and concentration of total cholesterol (a), LDL cholesterol (b), HDL cholesterol (c), and triglycerides (d) in training and control groups.

In the control group, we found positive significant relationships between postexercise irisin change and LDL and total cholesterol. Surprisingly, in the training group, the relationships between irisin change and lipids parameters were also significant but inverse (Figure 3). Neither in the training nor in the control groups was the irisin change significantly correlated with BMI (*R* = −0.24 and *p* = 0.57; *R* = 0.21 and *p* = 0.52, respectively), VO_2 max_ (*R* = −0.07 and *p* = 0.87; *R* = −0.07 and *p* = 0.83), concentration of glucose (*R* = 0.17 and *p* = 0.67; *R* = −0.08 and *p* = 0.79), insulin (*R* = 0.19 and *p* = 0.65; *R* = 0.20 and *p* = 0.53), and HbA1C (%) (*R* = 0.46 and *p* = 0.25; *R* = 0.19 and *p* = 0.54).

## 4. Discussion

To the best of our knowledge, neither the acute changes in serum irisin concentrations after an exercise session in pregnant women nor the relationship between the acute irisin responses to prenatal exercise and lipids levels has been presented so far. Apart from our previous study [20], there are no published data on the irisin levels among women physically active during gestation.

Contrary to the findings by other authors who observed that the irisin levels rise throughout pregnancy [15–17], in our participants, the baseline irisin levels were not related to the week of gestation. We also noted that the preexercise irisin concentration was slightly and insignificantly lower in the training group compared to controls. This outcome corresponds to our previously published data that in regularly exercising pregnant women irisin levels in the 21^st^ and 29^th^ week of gestation were very similar; they even decreased in average by 3% despite the development of pregnancy [20]. We assume that prenatal physical activity removes the potential cause for higher secretion of irisin. There is a hypothesis that elevated circulating irisin is an adaptive response to compensate for the increasing insulin resistance typical for gestation and limit its adverse metabolic and vascular effects [16, 18]. Ebert et al. [11] observed that homeostasis model assessment of insulin resistance (HOMA-IR) remains as a positive predictor of irisin serum concentrations. Physical activity significantly decreases insulin resistance in pregnancy [32] and probably also the potential need for compensative irisin production [20]. Looking for a relationship between irisin concentrations and HOMA-IR or between irisin and irisin/HOMA-IR ratio suggested by Benedini et al. [14] in physically active pregnant women seems to be worth future research.

No significant difference in baseline irisin levels between the training and control groups is in line with the results of Benedini et al. [14], who also did not find significant differences in the basal concentrations of this hormone between groups of both sexes with various physical activity patterns: from elite athletes to individuals with sedentary lifestyle. Similar outcomes in healthy women were obtained in other studies [20, 33]. However, in a few works, higher physical activity was associated with lower circulating irisin [8, 34, 35]. Therefore, the discussion on the effect of regular exercise on baseline irisin levels is still open [36]. Based on meta-analysis by Qiu et al. [37], regular resistance training seems to have more impact on decreasing irisin concentration in blood compared to endurance training.

A very important outcome of this study is that after an exercise session the irisin levels increased significantly in the training pregnant women. Based on the review by Fatouros [36], acute exercise protocols are potent stimuli for irisin release if they are characterized by adequate duration (>45 min) and/or intensity (>60% of maximal oxygen consumption or VO_2 max_). We applied an exercise session meeting above requirements.

We were aware of the lack of data on the postexercise irisin change in pregnancy. Therefore, in order to discuss our results, we had looked at least for the research performed in women or in groups combining both sexes. The observed 14% rise in irisin concentration after acute exercise in the training group corresponded to the increase of this hormone observed in other studies: 11% by Huh et al. [8], 12% by Winn et al. [38], and 23% by Kraemer et al. [39]. Nygaard et al. [40] and Zügel et al. [41] found higher postexercise release of irisin, as well. However, we should be cautious discussing our data with the results of the above works because the study women were obese [38], much younger or older [8], or with much higher levels of exercise capacity [39, 40]. In addition, the applied exercise protocols differed in type, intensity, and volume, which makes discussion with their results difficult.

An interesting outcome is that in all participants from the training group we observed the postexercise rise in irisin. Conversely, the controls responded in diverse ways: from irisin drop to higher secretion of this hormone than observed in the training group. It is not inconceivable that the training women after a few weeks of attending structured exercise sessions were more skilled in keeping the desired intensity, this being similar for all individuals within this group. Although we instructed the controls how to use the exercise options according to instructor's commands, heart rate monitors, RPE scale, and the talk test, employing them for the first time could be difficult and they might have failed to keep proper intensity. Some of the control women could have exercised more intensely than the training group, which could cause the higher rise in postexercise irisin. In turn, these five controls, who presented irisin drop, might have exercised less intensely. Caution regarding too high intensity is typical for pregnancy [42] and it would be not surprising regarding women who participated in this exercise session for the first time. Fatouros pointed out in his review [36] that most protocols of moderate intensity (≤60% VO_2 max_) failed to alter irisin status. On the other hand, the simultaneous use of four independent methods of monitoring the intensity of the exercise session (HR monitors, RPE scale, talk test, and instructor's observation and advice) was intended to provide both groups with similar training stimuli. Thus, the variety of irisin responses in the control group may reflect different metabolic reactions in nontraining pregnant women after a single exercise bout. They can be determined by their different physical activity patterns before the experiment.

According to a recent meta-analysis by Fox et al. [43], the rise of circulating irisin after exercise was related to participants' fitness levels. In contrast to their outcomes, in our study, the values of VO_2 max_ were not correlated with postexercise irisin changes in any of the group. However, our results may be affected by the progress of gestation. A decrease of VO_2 max_ throughout pregnancy was reported in previous studies [44, 45]. In general population of women, Chakaravertty et al. [45] observed that cardiorespiratory fitness was reduced by 6%, 9%, and 18% in the first, second, and third trimesters, respectively, when compared to the values of nonpregnant controls. We did not report such significant changes in exercise capacity related to the trimesters of pregnancy. It may be a positive adaptive effect of 6-week exercise program in the training group. The training women, who were in a few week older pregnancies, presented slightly lower exercise capacity levels compared to controls. Nonetheless, this difference between groups was neither of the statistical nor of clinical significance.

Some authors found that in the general population of pregnant women the baseline irisin was positively correlated with total cholesterol [18, 19] and also with low-density lipoproteins, high-density lipoproteins, and triglycerides [18]. However, they had not presented the physical activity level of their study participants. In contradiction to these results, in our previous study in regularly exercising pregnant women, we observed the tendency towards inverse relationships of baseline irisin with serum lipids, but they were statistically insignificant [20]. Benedini et al. [14] found a significant inverse correlation between blood lipids and baseline irisin reveals after enough total training load—in their study, it was at least 4 times a week and for a total of at least 10 hours per week. Taking into account that the exercise has more acute than chronic effect on irisin levels [36], in this work, we aimed to analyze the relationship between blood lipids and postexercise change in irisin. We found surprising results because in controls these relationships were positive and in the training group—inverse.

In the control group, the higher cholesterol levels the women had, the more irisin they secreted 30 minutes after a single exercise session. The lower the cholesterol levels, the lower was the postexercise irisin release. We might speculate that in pregnant women there might be a physiological defense mechanism protecting them from excessive inhibition of cholesterol synthesis to keep its optimal level for proper pregnancy development. The defense mechanism could show itself for instance in blocking the postexercise production of irisin, if necessary, in order not to activate AMPK. Although pathological dyslipidemia may lead to complications during gestation [6], extremely low TC, HDL, and LDL may increase the risk of preterm delivery [46].

In the training group, higher postexercise release of irisin was significantly related to lower total cholesterol. It must be underlined that in all training women the lipid concentrations were within reference values. As Ramírez-Vélez et al. [2] noted, prenatal physical activity allows to balance pregnancy-induced blood lipid gain. Considering irisin as a regulator of lipid metabolism [36] and based on the findings by other authors and our results, it is tempting to conclude that systematic participating in a structured prenatal physical activity program optimizes the postexercise irisin secretion. It can be related to more favorable utilization of lipids during physical effort, which is a typical adaptive mechanism after regular submaximal exercise [47].

Many authors have shown a positive correlation between circulating irisin and BMI in various populations of pregnant [18] and nonpregnant [9, 34, 48, 49] women. Based on the meta-analysis including studies with female participants, Fox et al. [43] reported that individuals with higher BMI presented lower immediate postexercise change in irisin. They assumed that the adiposity levels might alter basal irisin levels, which could impact the exercise response and irisin concentration during and after exercise. So far, there are no data on the relationship between BMI and the acute postexercise change in this hormone in pregnant women. In our study, in neither of the groups, we reported a correlation between study participants' BMI and the change in irisin levels 30 minutes after a single exercise session. This may be due to the fact that all tested women had normal prepregnancy BMI and normal gestational weight gain, which might be insufficient to impact the postexercise concentration of irisin. Although BMI levels varied between groups on the day of the experiment, this difference was related to the stage of pregnancy, not to the overweight or obesity. Therefore, we concluded that the diverse responses between groups in acute postexercise changes in irisin were affected by factors others than BMI (e.g., participants' previous physical activity patterns or exercise capacity). Nevertheless, based on the studies by other authors, we can speculate that pregnant women with different adiposity levels may secrete different amounts of irisin during and after exercise. This issue is worth future research, especially among obese pregnant women.

Such experimental studies should be conducted in larger groups, as the small sample size of this study is one of its weak points. We are aware that we should be cautious about generalizing conclusions based on data collected from 20 individuals. However, the obtained results show a clear tendency to diverge postexercise change in irisin concentration between training and nontraining pregnant women. Analyzing our data, we had in mind that antibodies currently used in ELISA kits for irisin detection may seriously overestimate its concentration [36]. It would be worthwhile to measure irisin concentration in physically active pregnant women using mass spectrometry.

Certainly, above interpretations require more in-depth analysis. Following Fatouros' suggestions [36] and work by Huh et al. [8], a wider approach for experimental exercise studies measuring not only circulating irisin would be desirable. Other proteins that may be connected to irisin like peroxisome proliferator-activated receptor-*γ* coactivator 1 (PGC-1*α*), fibronectin type III domain-containing protein 5 (FNDC5), uncoupling protein 1 (UCP1), and AMP should be explored in pregnant women participating in various exercise modes.

One of the study limitations is that we collected blood only at one time point after exercise. We have chosen such a study procedure based on available scientific evidence, including the paper by Huh et al. [9] reporting on the upregulation of irisin 30 minutes postexercise. Subsequent work has shown that circulating irisin increased immediately after high-intensity interval exercise, continuous moderate-intensity exercise, and resistance exercise sessions and declined 1 hour later [50]. Winn et al. [38] reported that irisin levels remained elevated during resting for 125 minutes after exercise of moderate intensity, whereas irisin levels returned to baseline within 15 minutes after high-intensity exercise. In a recent study, Qiu et al. [51] observed that irisin remained elevated 10 minutes after 50 min cycling at 80% of VO_2 max_ and 10 minutes after graded running to exhaustion; however, it decreased towards baseline levels 10 minutes after graded cycling to exhaustion. Therefore, for the future studies, it would be valuable to collect blood samples in a series of time points in order to depict the kinetics of irisin response to acute exercise in pregnancy, e.g., during the exercise, immediately after the end of the exercise session, and then 10, 30, 60, and 120 minutes postexercise. Comparison of the effects of exercise sessions with different intensity and modes also seems interesting.

Among other limitations of our work is the specificity of the controls. First, for organizational reasons, the control women were in lower pregnancy by an average of seven weeks compared to the training group. Nevertheless, the baseline irisin levels in both groups were not significantly different. Second, the controls were healthy and fit women, interested in exercise. Both groups on the initial assessment reported that they had met recommended level of physical activity before pregnancy (which was one of the inclusion criteria to our experiment). The distinction between groups was that the control women had not participated in any structured exercise program from conception to the study enrolment. It is probable that their spontaneously undertaken exercise during this period could influence their irisin response to physical effort. A detailed analysis of their physical activity patterns would be valuable here.

In future research, it would be also interesting to compare irisin levels and their changes after a single exercise bout between women who were active and inactive, both before and during pregnancy. However, in this case, the exercise intervention should be well balanced so as not to exceed exercise capacities of inactive individuals but remain a sufficient training stimulus for regular exercisers. For future studies, the randomized controlled trial design should be considered. Obviously, for ethical reasons, we would not randomize women to the training and not training groups, as the inactivity during pregnancy is unfavorable for the health of the mother and child. We could randomly subject a pregnant woman to different exercise interventions, e.g., continuous vs. interval modes. Such research could properly guide the development of widely available prenatal physical activity programs.

## 5. Conclusions

Training and nontraining (control) pregnant women responded differently to a single exercise session, both in the irisin change 30 minutes after exercise and its relationship to the blood lipids. Only in the training group, the postexercise increase was related to more favorable lipid profile. We are tempted to conclude that structured systematic physical activity in pregnancy may optimize the postexercise irisin response and lipid metabolism regulated by this hormone. Therefore, prenatal exercise programs should be promoted among pregnant women and obstetric care providers. Taking into account the beneficial postintervention effect on the irisin levels and the fact that we have observed no adverse outcomes in any of our participants, one of the proposals for pregnant women may be a program based on such exercise sessions as used in this study.

## Figures and Tables

**Table 1:** Characteristics of the study participants.

Variable	All pregnant women *n* = 20	Training group *n* = 8	Control group *n* = 12	*p* value
Age (y)	30 ± 3	31 ± 4	30 ± 3	0.39^∗^
Gestational age (wk)	28 ± 6	32 ± 5	25 ± 3	0.009^∗^
BMI (kg · m^−2^)	23.4 ± 2.5	25.2 ± 2.1	22.0 ± 2.1	0.007^∗^
VO_2 max_ (ml·kg^−1^·min^−1^)	23 ± 3.7	22.0 ± 2.8	23.3 ± 4.3	0.46^∗^
*HR zones for exercise sessions*				
HR lower limit (b·min^−1^)	126 ± 12	132 ± 12	123 ± 11	0.09^∗^
HR upper limit (b·min^−1^)	148 ± 13	151 ± 10	146 ± 14	0.38^∗^

BMI: body mass index; VO_2 max_: maximal oxygen capacity; HR: heart rate. ^∗^Student's *t*-test; values are presented as means ± SD; *p* ≤ 0.05 was considered statistically significant.

**Table 2:** Selected blood parameters at baseline.

Blood parameters	All pregnant women *n* = 20	Training group *n* = 8	Control group *n* = 12	*p* value
Irisin (ng·ml^−1^)	13.2 ± 3.0	12.2 ± 2.4	13.9 ± 3.3	0.23^∗^
TG (mg·dl^−1^)	162 ± 50	190 ± 56	144 ± 39	**0.04** ^∗^
TC (mg·dl^−1^)	276 ± 53	306 ± 40	256 ± 52	**0.03** ^∗^
LDL (mg·dl^−1^)	149 ± 36	170 ± 28	135 ± 34	**0.03** ^∗^
HDL (mg·dl^−1^)	95 ± 21	97 ± 21	93 ± 23	0.67^∗^
Insulin (ng·ml^−1^)	3.5 (1.2-13.7)	3.2 (2.0-6.4)	3.8 (1.2-13.7)	0.94^∗∗^
Glucose (ng·ml^−1^)	81 ± 6	79 ± 6	81 ± 6	0.5^∗^
HbA1c (%)	4.8 ± 0.26	4.9 ± 0.2	4.7 ± 0.3	**0.05** ^∗^

TG: triglycerides; TC: total cholesterol; LDL: low-density lipoproteins; HDL: high-density lipoproteins; HbA1c: glycated hemoglobin. Values are presented as means ± SD or as median (25^th^ and 75^th^ percentiles); ^∗^Student's *t*-test; ^∗∗^Mann-Whitney *U* test; *p* ≤ 0.05 was considered statistically significant.

## Data Availability

The full trial protocol and raw data supporting the conclusions of this manuscript will be made available by the authors, without undue reservation, to any qualified researcher.
